# Emerging roles of micronuclei: Neuronal micronuclei regulate microglial properties

**DOI:** 10.4103/NRR.NRR-D-25-01470

**Published:** 2026-02-05

**Authors:** Chihiro Maeda, Fuminori Tsuruta

**Affiliations:** Doctoral Program in Biology, Degree Programs in Life and Earth Sciences, Graduate School of Science and Technology, University of Tsukuba, Tsukuba, Ibaraki, Japan; Master’s and Doctoral Programs in Biology, Institute of Life and Environmental Sciences, University of Tsukuba, Tsukuba, Ibaraki, Japan; Ph.D. Program in Human Biology, Graduate School of Comprehensive Human Sciences, University of Tsukuba, Tsukuba, Ibaraki, Japan; Ph.D. Program in Humanics, School of Integrative and Global Majors, University of Tsukuba, Tsukuba, Ibaraki, Japan; Master’s and Doctoral Program in Neuroscience, Graduate School of Comprehensive Human Sciences, University of Tsukuba, Tsukuba, Ibaraki, Japan; Center for Quantum and Information Life Sciences, University of Tsukuba, Tsukuba, Ibaraki, Japan

Microglia are the resident immune cells of the brain, playing indispensable roles in maintaining cerebral homeostasis from embryonic development through aging (Prinz et al., 2019). Beyond their classical immune functions, microglia are now recognized as active participants in a wide range of processes, including synaptic pruning, clearance of apoptotic neurons, neurogenesis, and synaptogenesis (Borst et al., 2021). Recent fate mapping analyses have identified that microglia originate from the yolk sac and migrate into the brain primordium (Ginhoux et al., 2010). After migration, microglia colonize the embryonic brain primordium, proliferate before and after birth, and subsequently persist as long-lived self-renewing cells within the central nervous system (Barry-Carroll et al., 2023). Once established, they undergo context-dependent state transitions characterized by distinct transcriptional profiles, thereby acquiring diverse properties and functions (Masuda et al., 2020). Recent advances in single-cell transcriptomics and *in vivo* imaging have further revealed remarkable heterogeneity among microglial populations. Due to this unexpected complexity, the definition of microglial heterogeneity is currently under discussion (Paolicelli et al., 2022). These specialized populations exhibit unique anatomical niches and functional repertoires, which contribute to immune surveillance, vascular integrity, and the maintenance of the blood–brain barrier. Although this diversity is thought to be shaped by a variety of signals within the brain environment, the key factors underlying such heterogeneity have remained elusive. Recently, we discovered novel mechanisms underlying the regulation of microglial properties by micronuclei (MNs) (Yano et al., 2025). In this perspective, we present our findings demonstrating that MNs act as potential mediators of neuron-to-microglia communication and consequently regulate microglial diversity.

First, we observed the presence of MNs in the superficial layers during our observation of the cerebral cortex at embryonic day 18.5. To investigate the mechanism underlying the generation of MNs, we introduced an enhanced green fluorescent protein (EGFP)-expressing plasmid into neural stem cells along the lateral ventricle using *in utero* electroporation. EGFP-labeled migrating neurons were squeezed within the primitive cortical zone, a region of high cell density, and subsequently produced MNs. We also found that neurons passing through the small pore produce MNs by an *in vitro* trans-well assay.

Although the possibility that other effects, such as cell-cell interactions and extrinsic factors, cannot be excluded, we propose that physical stress during neuronal migration through narrow regions induces the formation of MNs. We next asked whether neuronal MNs are released extracellularly. Both *in vitro* and *in vivo* analyses confirmed the existence of MNs outside neurons, some of which were surrounded by membrane-like structures, indicating secretion as extracellular vesicles. As inhibition of lysosomal activity increased the amount of extracellular MNs, we presumed that MNs secretion is involved in the autophagy-dependent secretion pathway. Liquid chromatography-mass spectrometry analysis revealed that MNs contain tubulins, Rab family proteins, and T-complex protein Ring Complex proteins. Notably, T-complex protein Ring Complex proteins function as an adaptor that links target proteins with LC3 and promotes autophagosome formation. Thus, it is likely that neuronal MNs are secreted as extracellular vesicles through an autophagy-related pathway (Asami et al., 2025). Next, we investigated whether the propagate of MNs occurs in the brain. We analyzed the NexCre; LSL-SUN1-GFP mice, in which the nuclear envelope of neurons is specifically labeled with GFP. We observed neuronal MNs in the superficial area, some of which were taken up by microglia. Importantly, MN-harboring microglia exhibit a decrease in the length and number of their processes, suggesting that neuronal MNs are transferred to microglia and alter their morphology. To confirm this phenomenon, we conducted two-photon *in vivo* imaging analysis using Cx3cr1^EGFP/+^; H2B-mCherry mice. After incorporating MNs, microglial processes were gradually shortened. Moreover, some microglia incorporating MN extended processes toward the brain surface and migrated in that direction. Because MNs are recognized by the cytosolic DNA sensor cyclic GMP-AMP synthase (cGAS), we examined whether cGAS is necessary for MN-dependent microglial changes. The loss of cGAS abolished MN-dependent morphological changes, demonstrating that cGAS is required for MN-induced remodeling of microglial properties. To assess transcriptional changes, we purified CD11b^+^ GFP^±^ microglia from NexCre: LSL-Sun1-GFP mice and performed bulk RNA sequencing. MN^+^ (GFP^Low^) microglia exhibited upregulation of extracellular matrix-related genes and shared signatures with central nervous system-associated macrophages, including expression of CD206, Dab2, Stab1, and Slc40a1. Collectively, these data indicate that neuronal MNs alter the transcriptional profile of microglia.

This study demonstrates that neuronal MNs are released into the extracellular space, incorporated by microglia, and change microglial characteristics (**Figure 1**). Nevertheless, several questions remain to be addressed. First, the precise signaling pathways by which MNs alter microglial properties remain incompletely defined. We found the involvement of the cGAS signaling by MN incorporation. The cGAS–STING pathway in microglia is known to be involved in several neurodegenerative disorders through aberrant activation of inflammatory signaling (Xie et al., 2023). While we found that the cGAS axis is involved in changes in microglial properties, we did not observe significant induction of interferon γ following MN incorporation. Therefore, it may be necessary to consider the possibility that other pathways, beyond the canonical cGAS–STING–IRF3 axis, are involved in changes in microglial properties during developmental stages. The functions of cGAS underlying the regulation of microglial characteristics seem to be distinct from the pathological conditions. Second, the molecular mechanisms of MNs propagation also require further investigation. We observed that autophagy-dependent secretion pathways are involved in MNs propagation; however, we have not obtained compelling evidence using *in vivo* imaging analysis or other methods. Although the mechanism by which microglia take up MNs is currently thought to involve membrane fusion between the plasma membrane and the membrane of an extracellular vesicle, we cannot exclude the possibility that microglia internalize MNs by phagocytic pathway. To further clarify these phenomena, cellular biological approaches, in addition to *in vivo* analyses, are considered important. Lastly, the physiological significance of MNs propagation is one of the most intriguing questions. So far, we have found that neuronal MNs are present in the meninges (Yano et al., 2025). We also found that MN propagation occurs not only in developmental stages but also in the aged brain. In the aging stage, MN-harboring microglia extend processes to blood vessels. Microglia are thought to be actively involved in repairing and reinforcing damaged blood vessels, and conversely, in inducing blood–brain barrier dysfunction (Maeda and Tsuruta, 2024). Thus, we presume that microglia incorporating MNs change their own characteristics and regulate vascular systems and the overall brain environment. Comparing MN-harboring microglia between developmental stages and aging contexts represents an important and intriguing future direction.

**Figure 1:**
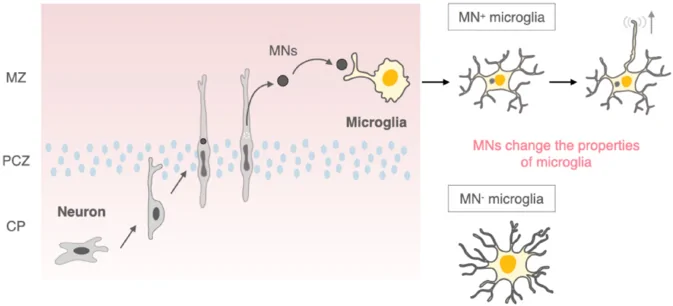
Neuronal MNs contribute to the regulation of microglial properties during brain development. Physical stress associated with neuronal migration induces the formation of MNs, which are subsequently incorporated by microglia. Following uptake, microglia exhibit alterations in their morphology and transcriptional programs. CP: Cortical plate; MZ: marginal zone; PCZ: primitive cortical zone.

In conclusion, this study highlights a previously unrecognized role of neuronal MNs as mediators of intercellular communication. Physical stress generated as migrating neurons pass through restricted regions has been shown to induce the formation of neuronal MNs. These neuronal MNs are subsequently incorporated by microglia. Transcriptomic analyses further suggest that MNs reprogram the transcriptional states of microglia, thereby contributing to the modulation of their functional properties. Collectively, these findings provide a new conceptual framework in which neuronal MNs act as niche-derived signals shaping microglial states during brain development. In the future, we aim to elucidate the subsequent functions of MN-incorporating microglia during development, as well as to clarify the interactions between neuronal MNs and microglia in the aging brain, with the goal of uncovering the mechanisms linking them to disease relevance and therapeutic strategies.

## References

[R1] Asami N, Yano S, Tsuruta F (2025). Potential for micronuclear turnover through autophagy secretion pathway. MicroPubl Biol.

[R2] Barry-Carroll L, Greulich P, Marshall AR, Riecken K, Fehse B, Askew KE, Li K, Garaschuk O, Menassa DA, Gomez-Nicola D (2023). Microglia colonize the developing brain by clonal expansion of highly proliferative progenitors, following allometric scaling. Cell Rep.

[R3] Borst K, Dumas AA, Prinz M (2021). Microglia: immune and non-immune functions. Immunity.

[R4] Ginhoux F, Greter M, Leboeuf M, Nandi S, See P, Gokhan S, Mehler MF, Conway SJ, Ng LG, Stanley ER, Samokhvalov IM, Merad M (2010). Fate mapping analysis reveals that adult microglia derive from primitive macrophages. Science.

[R5] Maeda C, Tsuruta F (2024). Molecular basis of neuronal and microglial states in the aging brain and impact on cerebral blood vessels. Int J Mol Sci.

[R6] Masuda T, Sankowski R, Staszewski O, Prinz M (2020). Microglia heterogeneity in the single-cell era. Cell Rep.

[R7] Paolicelli RC (2022). Microglia states and nomenclature: a field at its crossroads. Neuron.

[R8] Prinz M, Jung S, Priller J (2019). Microglia biology: one century of evolving concepts. Cell.

[R9] Xie X, Ma G, Li X, Zhao J, Zhao Z, Zeng J (2023). Activation of innate immune cGAS-STING pathway contributes to Alzheimer’s pathogenesis in 5xFAD mice. Nat Aging.

[R10] Yano S (2025). Propagation of neuronal micronuclei regulates microglial characteristics. Nat Neurosci.

